# Differential expressions of anthocyanin synthesis genes underlie flower color divergence in a sympatric *Rhododendron sanguineum* complex

**DOI:** 10.1186/s12870-021-02977-9

**Published:** 2021-04-28

**Authors:** Lin-Jiang Ye, Michael Mӧller, Ya-Huang Luo, Jia-Yun Zou, Wei Zheng, Yue-Hua Wang, Jie Liu, An-Dan Zhu, Jin-Yong Hu, De-Zhu Li, Lian-Ming Gao

**Affiliations:** 1 CAS Key Laboratory for Plant Diversity and Biogeography of East Asia, Kunming Institute of Botany, Chinese Academy of Sciences, Kunming, 650201 Yunnan China; 2 Germplasm Bank of Wild Species, Kunming Institute of Botany, Chinese Academy of Sciences, Kunming, 650201 Yunnan China; 3 School of Life Sciences, Yunnan University, Kunming, 650091 Yunnan China; 4 University of Chinese Academy of Sciences, Beijing, 10049 China; 5 Royal Botanic Garden Edinburgh, Edinburgh, EH3 5LR UK; 6 Yunnan Lijiang Forest Ecosystem National Observation and Research Station, Kunming Institute of Botany, Chinese Academy of Sciences, Lijiang, 674100 Yunnan China

**Keywords:** Anthocyanin synthesis, Comparative transcriptomics, Flower coloration, Gene expression, *Rhododendron sanguineum* complex, Sympatric speciation

## Abstract

**Background:**

The *Rhododendron sanguineum* complex is endemic to alpine mountains of northwest Yunnan and southeast Tibet of China. Varieties in this complex exhibit distinct flower colors even at the bud stage. However, the underlying molecular regulations for the flower color variation have not been well characterized. Here, we investigated this via measuring flower reflectance profiles and comparative transcriptome analyses on three coexisting varieties of the *R. sanguineum* complex, with yellow flush pink, bright crimson, and deep blackish crimson flowers respectively. We compared the expression levels of differentially-expressed-genes (DEGs) of the anthocyanin / flavonoid biosynthesis pathway using RNA-seq and qRT-PCR data. We performed clustering analysis based on transcriptome-derived Single Nucleotide Polymorphisms (SNPs) data, and finally analyzed the promoter architecture of DEGs.

**Results:**

Reflectance spectra of the three color morphs varied distinctively in the range between 400 and 700 nm, with distinct differences in saturation, brightness, hue, and saturation/hue ratio, an indirect measurement of anthocyanin content. We identified 15,164 orthogroups that were shared among the three varieties. The SNP clustering analysis indicated that the varieties were not monophyletic. A total of 40 paralogous genes encoding 12 enzymes contributed to the flower color polymorphism. These anthocyanin biosynthesis-related genes were associated with synthesis, modification and transportation properties (*RsCHS*, *RsCHI*, *RsF3H*, *RsF3*′*H*, *RsFLS*, *RsANS*, *RsAT*, *RsOMT*, *RsGST*), as well as genes involved in catabolism and degradation (*RsBGLU*, *RsPER*, *RsCAD*). Variations in sequence and *cis*-acting elements of these genes might correlate with the anthocyanin accumulation, thus may contribute to the divergence of flower color in the *R. sanguineum* complex.

**Conclusions:**

Our results suggested that the varieties are very closely related and flower color variations in the *R. sanguineum* complex correlate tightly with the differential expression levels of genes involved in the anabolic and catabolic synthesis network of anthocyanin. Our study provides a scenario involving intricate relationships between genetic mechanisms for floral coloration accompanied by gene flow among the varieties that may represent an early case of pollinator-mediated incipient sympatric speciation.

**Supplementary Information:**

The online version contains supplementary material available at 10.1186/s12870-021-02977-9.

## Background

The remarkable diversity of flower colors, especially in wild plants has fascinated botanists, ecologists, and horticulturists for centuries [1–3]. The coloring of floral organs, a remarkable character of flowering plants, is a striking feature of the angiosperm radiation [4, 5]. Flower color diversity is recognized to be one of key adaptive traits correlated predominantly with pollinators (e.g. insects, birds) and animals for seed dispersal [6, 7]. Moreover, the flower color phenotype is an important feature for plants used for their classification by taxonomists. However, flower color appears evolutionarily to be one of the most labile traits, down to populations in the same species [7, 8].

The cellular compounds of flowers that contribute to the color profile and visually perceived by humans are generally referred to as “pigments”. A group of secondary metabolites belonging to flavonoids are the main determinants of pigments for coloration in plants, where anthocyanins are responsible for red orange to red, purple to violet pigments found in flowers, leaves, fruits, seeds and other tissues [9, 10]. Anthocyanins are the predominant compounds of floral coloration, existing in over 90% of angiosperms [11]. The flavonoid biosynthetic pathway leading to accumulation of anthocyanins is highly conserved and well characterized, and has been extensively studied in many species, most of which are in model plants or agriculturally and horticulturally important plants [12–15]. Few studies have examined the molecular basis underlying the formation and accumulation of anthocyanin in wild species [16, 17]. Based on these studies, three major associated factors have been proposed to be involved in anthocyanin accumulation, including transcription regulatory genes (MYB-bHLH-WD40 complex) that occur in the nucleus, structural genes (*CHS*, *FLS*, *DFR, ANS*) acting in the biosynthetic pathway, and transporter genes (*GST*) transferring anthocyanin from the cytosol into the vacuole [10, 18, 19]. The expression of these genes could also be affected by natural variation in sequences and *cis*-regulatory elements as well as epigenetic modifications (such as DNA methylation) in the promoter regions [18, 20]. Moreover, the color of flowers can be stabilized and enhanced by co-pigmentation of anthocyanins by flavonols, where it is observed as hyperchromic effect, in which the intensity of an anthocyanin content is fortified [21]. For instance, the *DFR* gene along with the *FLS* gene can compete for a substrate leading to the production of different anthocyanins and flavanols through two primary branches [22, 23], thus resulting in co-pigmentation. In contrast to the biosynthesis pathways, knowledge of anthocyanin catabolism in plants is limited. Some catabolic genes like *BGLU* and *PER* have been shown to be responsible for anthocyanin degradation [24, 25]. Nevertheless, the molecular mechanism regulating anthocyanin synthesis has been shown to vary among plant species resulting in structural diversity of anthocyanins, because the biosynthesis pathway is regulated by multiple factors through regulatory networks [26].

Color is a form of electromagnetic radiation in the range of the visible spectrum. The wavelengths reflected by pigments determine the color of a flower [27]. Color can be defined and classified in terms of Brightness (the intensity of a signal, B), Saturation (the purity of a color, S) and Hue (the spectral descriptor of color, H), and those features are commonly used to distinguish colors [27, 28]. Brightness refers to the color intensity that is determined by the amount of anthocyanin [29, 30], and different color component combinations such as B/H, S/H were found to be significantly correlated with anthocyanin content as well [31]. Liu et al. [32] proposed that the color brightness decreased as the total anthocyanin content increased. It was also demonstrated that a correlation exists between the saturation/hue ratio (S/H) and anthocyanin content [31]. With these parameters, the anthocyanin content can be rapidly and non-destructively determined.

In evergreen azaleas (*Rhododendron*), anthocyanins and flavanols are the main flower pigments, and especially the composition of anthocyanin constituents (i.e. cyanidin, delphinidin, malvidin, pelargonidin, peonidin, and petunidin), and their quantities determine their flower color that ranges from light pink to violet [11, 33]. Some studies have reported that *R. kiusianum* with purple-colored flowers contain derivatives of both anthocyanidins cyanidin and delphinidin, whereas the red-colored flowers of *R. kaempferi* contain only cyanidin derivatives [34]. Le Maitre et al. [35, 36] studying *Erica* species, belonging to the same family Ericaceae as *Rhododendron*, used qRT-PCR and UPLC-MS, unraveled the anthocyanin genetic network of floral color shifts between red or pink and white or yellow flowered species and found losses of single pathway gene expression, abrogation of the entire pathway due to loss of the expression of a transcription factor or loss of function mutations in pathway genes resulted in striking floral color shifts.

Here, we investigated the genetic basis of flower coloration using a highly color polymorphic *Rhododendron sanguineum* complex. The complex (*R*. subgen. *Hymenanthes*) includes plants with yellow to pink or crimson to blackish crimson flowers that are classified into six varieties mainly based on their flower color differences [37]. Members of this complex are basically located at high elevations (> 3000 m) associated with snow cover [37]. They are endemic to northwest Yunnan and southeast Tibet, one of the global biodiversity hotspots [38]. This region is also recognized as one of the centers for diversification and differentiation of *Rhododendron* [37, 39]. The flower color polymorphisms of this genus have been traditionally viewed as an ecologically adaptive trait that is essential in attracting specific pollinators [40–42], and may also be the response to environmental variation, such as UV radiations at different elevations, temperatures, and soil conditions [32]. Although there are studies published on the anthocyanin components and contents in *Rhododendron* flowers, most were solely dedicated to the identification of the pigment constituents in the petals of some wild and cultivated azaleas using thin-layer chromatography (TLC) and high-performance liquid chromatography (HPLC) [11, 33]. No study so far focused on the molecular mechanisms underlying infraspecific color polymorphisms in *Rhododendron*. The study of closely related entities such as a species complex has the advantage of a fairly homogeneous genetic background where flower color genes vary and cases of homoplasy are limited. Previous studies mainly focused on color shifts at different developmental stages of a single species [14, 18], or covered a number of related species [26, 35].

In the present study, we combined transcriptome sequencing (RNA-seq) and genome resequencing with reflectance spectra analyses to elucidate molecular and anthocyanin content differences among three differently colored naturally occurring varieties of the *R. sanguineum* complex, with yellow flushed pink to deep blackish crimson colored flowers. We aimed at studying the correlation between infraspecific flower color variation and the expression of candidate genes of the anthocyanin / flavonoid biosynthesis pathway. Our findings may allow the proposal of a hypothesis for the genetic mechanism of the expression of flower color variation and a representative case of pollinator-mediated incipient sympatric speciation in the *R. sanguineum* complex. In addition, it is the first study to compare transcriptome profiles in a natural system of a non-model species of *Rhododendron*.

## Results

### Reflectance spectra and color morph differences

**Fig. 1:**
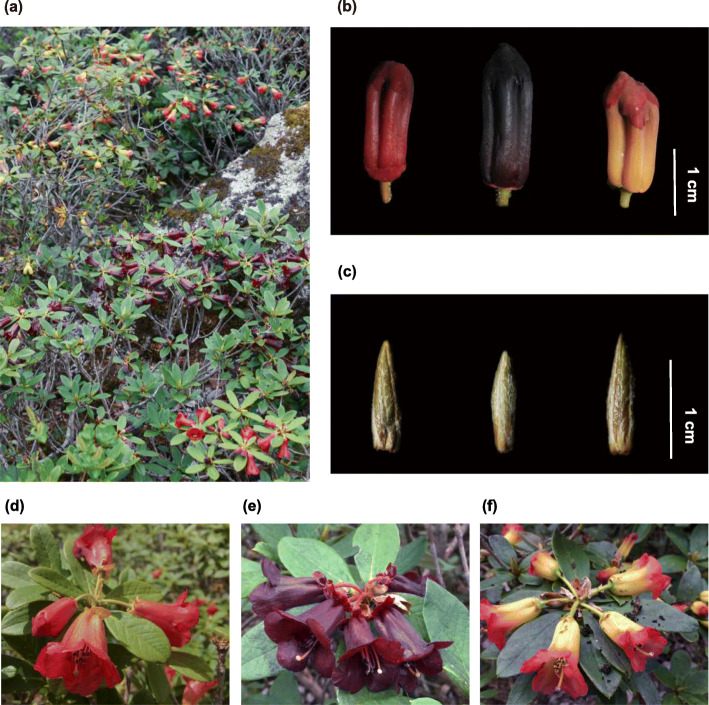
Morphology and sampling stages of the three different flower morphs found in the *Rhododendron sanguineum* complex in the wild. **a** Habitat of the sampling site; sampling stages of the two tissues for RNA-seq, including late flower bud (**b**) and leaf bud (**c**) of the varieties (left to right) *R. sanguineum* var. *sanguineum*, *R*. var. *haemaleum*, and *R*. var. *didymoides*; open flowers of *R.* var. *sanguineum* (**d**), *R.* var. *haemaleum* (**e**), and *R.* var. *didymoides* (**f**)

**Fig. 2:**
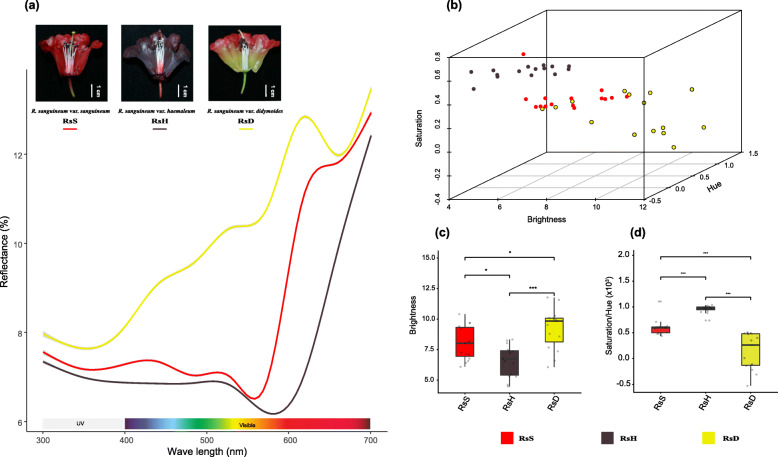
Comparisons of the reflectance spectra of three varieties of the *Rhododendron sanguineum* complex. **a** Reflectance spectra were measured at the midpoint of the corolla tubes of the three varieties. **b** Three-dimensional plots of the visible spectra that classified by brightness (x-axis), hue (y-axis), and saturation (z-axis) of the three varieties. **c** Boxplots of brightness values of the reflectance spectra of the three varieties. **d** Boxplots of the ratios of saturation over hue from reflectance spectra for the three varieties. Boxplots show the median, and the box edges represent the 25th and 75th percentiles of values for a total of 15 individuals for each variety. Statistical significance was determined by a two-tailed Student’s *t* test. The significant differences are noted as asterisks (*P < 0.05; **P < 0.01; ***P < 0.001). Red represents *R*. var. *sanguineum* (*RsS*); Purple represents *R*. var. *haemaleum* (*RsH*); Yellow represents *R*. var. *didymoides* (*RsD*)

The sampled individuals can be grouped into three color categories representing *R. sanguineum* var. *sanguineum* (*RsS*) with bright crimson flowers (Fig. 1d), *R.* var. *haemaleum* (*RsH*) where the flowers were deep blackish crimson (Fig. 1e), and *R.* var. *didymoides* (*RsD*) with yellow flushed pink tubes and red petals (Fig. 1f). The reflectance spectra of the three color morphs varied distinctively across the wavelength range, especially between 400 and 620 nm (Fig. 2a). *RsS* showed one discrete peak in the red spectrum (~ 620 nm), while *RsH* had a peak further into the far red range (> 700 nm). The reflectance of *RsD* increased across the blue to yellow spectrum and peaked in the orange-red range (~ 600 nm) (Fig. 2a). The flower colors of the three varieties exhibited marked differences in brightness, saturation and hue. *RsD* had the highest brightness values followed by *RsS*, and *RsH* with the lowest values, while *RsH* had the highest saturation, then *RsS* with *RsD* the lowest (Fig. 2b, c). The hue values were highest in samples of *RsD*, then *RsS* and *RsH* (Fig. 2b). The ratio of saturation and hue (an indirect measurement of anthocyanin content) of *RsH* was the highest compared to those of the *RsD* and *RsS* samples (Fig. 2d). The ratio values of the *RsD* samples varied much wider than those of the other two varieties that maybe linked to variation in the color composition of its bicolored flowers (Fig. 1).

### De novo transcriptome assembly and quality assessment

We sequenced a total of 18 RNA-seq libraries from two tissues (flower buds and leaf buds) for three individuals for each of the three varieties in the *R. sanguineum* complex using Illumina paired-end sequencing. After quality control, approximately 655.8 million (M) clean reads (~ 96 gigabase pairs, Gbp) remained with a very uniform number of reads between the libraries ranging between 32,111,674 and 43,353,842 (Table S1). The contig N50 values for the CORSET across the three varieties had similar lengths and ranged from 903 to 1125 base pairs (bp) and the numbers of transcripts ranged from 117,976 to 171,725 (Table S1). The reads of the 18 individual libraries were aligned by mapping the reads back to their variety-specific references, with mapping rates ranging from 84.25 to 94.85%: the average alignment rates were 92.82, 90.30 and 93.77% for *R.* var. *sanguineum*, *R.* var. *haemaleum* and *R.* var. *didymoides* respectively (Table S2). Based on the 1614 conserved BUSCO embryophyte orthologs, assessment of transcriptome completeness identified 1540 (95.4%) complete and fragmented BUSCOs in *R.* var. *sanguineum*, 1548 (95.9%) in *R.* var. *haemaleum* and 1538 (95.3%) in *R.* var. *didymoides* (Figure S1a, Table S3). Thus, our results indicated that the three transcriptomes were well assembled and relatively complete.

### Ortholog identification and functional annotation

The ORF prediction found a total of 53,207 protein coding transcripts among the final non-redundant transcripts for *R.* var. *sanguineum*, 46,754 for *R.* var. *haemaleum* and 38,548 for *R.* var. *didymoides*. For the three varieties, OrthoVenn2 [43] identified among the total of 138,509 protein coding transcripts 31,525 clusters, in which 16,361 were orthologous (containing at least two species) and 15,164 were single-copy genes. There were 54,192 singletons identified that were not included in any cluster (Table S4). Based on the annotation of the 15,164 one-to-one single copy orthologs (Table S5) among the three varieties, we found 14,441 (95.23%) matches in the National Center for Biotechnology Information non-redundant (NR) protein database, 10,648 (70.22%) in UniProt/Swiss-Prot, 13,089 (86.32%) in COG/KOG, 14,109 (93.04%) in eggNOG, 7445 (49.1%) in Gene Ontology (GO) and 4302 (28.37%) in the Kyoto Encyclopedia of Genes and Genomes (KEGG) (Figure S1b, Figure S2).

### SNP detection and clustering analyses

With the reference sequence set of 15,164 orthologous single-copy genes, the cDNA libraries of the 9 individuals of the *R. sanguineum* complex yielded a total of 50,853 SNPs. Based on this SNP data set, neither the SNPhylo tree nor the PCA analysis clustered the samples in variety-specific clusters (Figure S3). Only the *RsH* samples fell in one clade in the SNPhylo tree, but so did one sample of *RsD* (BS = 100%). *RsD* and *RsS* samples were intermixed in two clades.

### Overall differentially expressed genes and functional enrichment

**Fig. 3:**
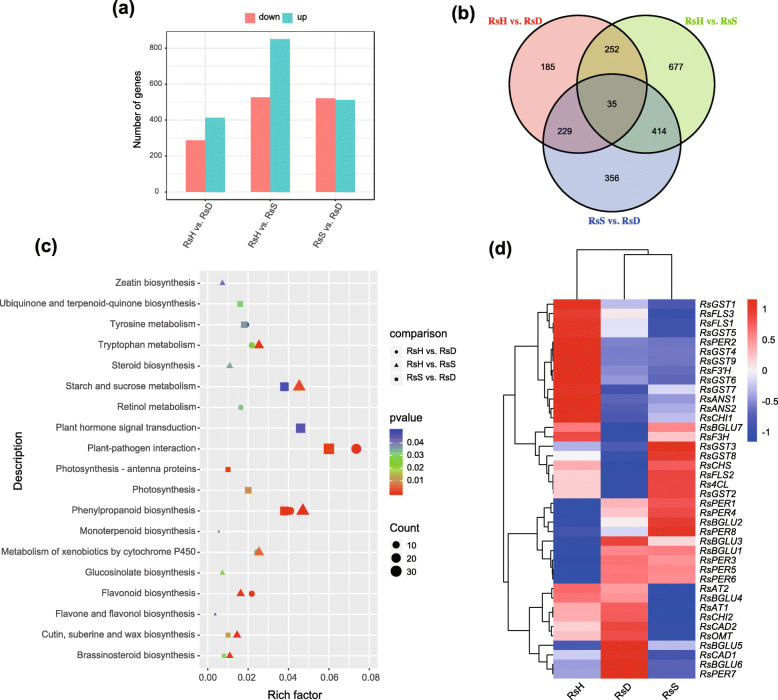
Results of the RNA-seq analyses of the three varieties of the *Rhododendron sanguineum* complex. Enrichment and Hierarchical clustering showing profiles of DEGs between *RsH* vs *RsD*, *RsH* vs *RsS*, and *RsS* vs *RsD*. **a** Histogram of differentially expressed genes via pairwise comparisons among the three varieties. **b** Venn diagram of differentially expressed genes. The sum of the numbers in the large section of each circle represents the total number of differentially expressed genes in pairwise variety comparisons; overlapping portions of the circles represent the differentially expressed genes shared between the compared varieties. **c** Scatterplot result of a KEGG pathway enrichment analysis of differentially expressed genes in three pairwise comparisons. Coloring of the *p*-values indicates the significance of the rich factor ranging from − 1 to 1. The rich factor is the ratio of DEG numbers annotated in a given pathway term to all gene numbers that were annotated in the pathway term, and greater rich factor values indicate greater intensiveness. The 19 top common pathway terms of three comparisons enriched in the KEGG database are listed in this figure. The size of the symbols represents the number count of DEGs. **d** Hierarchical clustering of normalized expression levels of all 40 candidate gene paralogs showing distinct gene expression profiles in pairwise comparisons of the *R. sanguineum* complex. Analyses were performed on the flower bud samples. Red indicates higher, while blue represents lower expression levels

We performed three pairwise transcriptome comparisons to identify DEGs among the flower color variants of the *R. sanguineum* complex by calculating the genes’ FPKM values. We detected a total of 2148 differentially expressed genes (DEGs) in the three-way comparisons between the varieties. In particular the comparison between the deep blackish crimson morph *RsH* with the yellow flushed pink morph *RsD* identified 701 DEGs (413 up-regulated and 288 down-regulated in *RsH*); for the comparison of *RsH* versus the deep blackish crimson morph *RsS*, 1378 DEGs were found (851 up-regulated and 527 down-regulated in *RsH*); the comparisons of *RsS* versus *RsD* gave 1034 DEGs (513 up-regulated and 521 down-regulated in *RsS*) (Fig. 3a, b). The differentially expressed genes were highest between *RsS* and *RsH*. All DEGs pattern are given in supplementary Figures S4 & S5. Based on the PCA and heatmap results of the gene expression profiles (Figure S4; Figure S6a), each variety clustered together (showing a species-specific pattern), which indicated that the RNA-seq libraries were reliable.

All DEGs were classified into three main categories of gene ontologies: biological processes (BP), cellular components (CC) and molecular functions (MF), in which most of the GO terms fell under biological processes, followed by molecular functions and cellular components. The three pairwise comparison results revealed that there were 40 common significant GO terms, including 27 BPs, 8 CCs and 5 MFs (Figure S6b, c). The genes from the different comparisons clearly indicated the same molecular and cellular events, such as plant resistance (GO:0006952, defense response) and metabolic process (GO:0009737, response to abscisic acid; GO:0009753, response to jasmonic acid). The KEGG pathway enrichment analysis showed that the DEGs were associated with various metabolic and biosynthesis pathways: 20, 17 and 29 pathways corresponding to 701, 1378 and 1034 DEGs were significantly enriched in *RsH* vs. *RsD*, *RsH* vs. *RsS*, and *RsS* vs. *RsD*, respectively. In particular, genes that encoded enzymes involved in phenylpropanoid biosynthesis (ko00940) were all significantly enriched. Furthermore, pathways related to flavonoid biosynthesis (ko00941) and metabolism of xenobiotics by cytochrome P450 (ko00980) were enriched in the comparisons of *RsH* vs. *RsD* and *RsH* vs. *RsS*. Some representative most significantly enriched KEGG pathways are shown in Fig. 3c. To gain more insights into the expression pattern of all DEGs, a heatmap was generated using the TMM normalized expression values (Figure S6a), here we focused on genes enriched in pigmentation-related terms.

### Candidate genes related to color polymorphism in the *R. sanguineum* complex

Based on the result of KEGG pathway enrichment of DEGs, and with consideration of removing extremely lowly expressed genes (FPKM < 1), we identified 13 candidate genes out of 40 paralogs that were putatively relevant for flower pigmentation, contributing to anthocyanin accumulation and/or co-pigmentation in this complex (Table S6). All were anthocyanin-associated genes involved in anthocyanin biosynthesis, anthocyanin modification and anthocyanin transport. These included anthocyanidin synthase (*ANS*), acyltransferase (*AT*), beta-glucosidase (*BGLU*), 4-coumarate-CoA ligase (*4CL*), cinnamyl alcohol dehydrogenases (*CAD*), chalcone isomerase (*CHI*), chalcone synthase (*CHS*), flavanone 3-hydroxylase (*F3H*), flavonoid 3′-hydroxylase (*F3*′*H*), flavonol synthase (*FLS*), glutathione S-transferase (*GST*), O-methyltransferase (*OMT*), and peroxidase (*PER*). Among these, *Rs4CL*, *RsCHS*, *RsF3H*, *RsF3′H* and *RsOMT* had only one copy, the others (*RsANS*, *RsAT*, *RsBGLU*, *RsCAD*, *RsCHI*, *RsFLS*, *RsGST*, and *RsPER*) represented multigene families with 2 (e.g. *RsAT*, *RsCAD*, *RsCHI*) to 8 or 9 copies (e.g. *RsBGLU*, *RsGST*, *RsPER*). In pairwise comparison analyses of those gene paralogs, 23 DEGs, 25 DEGs and 12 DEGs were identified in the pairs *RsH* vs. *RsD*, *RsH* vs. *RsS* and *RsS* vs. *RsD*, respectively (Fig. 3d; Figures S7, S8, S9). qRT-PCR confirmation to validate the reliability of candidate genes expression profiles of representative copies of the 13 candidate genes were obtained, except for *Rs4CL* that failed to amplify (Table S7).

**Fig. 4:**
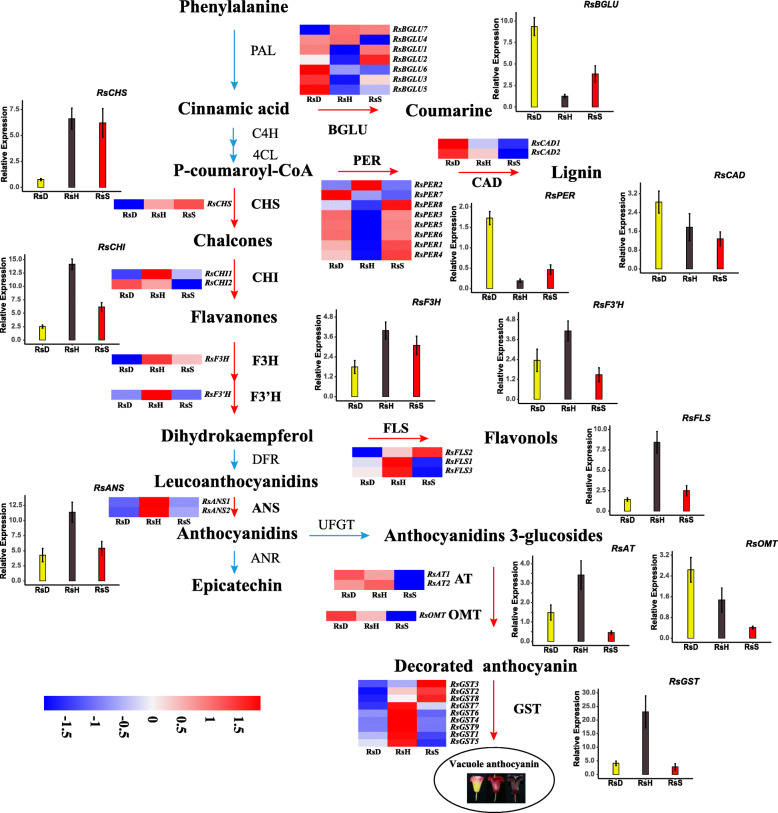
Schematic diagram of the flavonoid pathway related to flower pigmentation in the *Rhododendron sanguineum* complex. Enzyme acronyms, expression patterns, and qRT-PCR results are shown beside each metabolic step and direction of synthesis marked with red arrows. The RNA-seq expression pattern of each gene is shown in heatmaps. The color scale represents log2-transformed FPKM (fragments per kilobase of exon per million mapped reads) values. Red represents high expression, and blue represents low expression. *RsS*, *RsH*, *RsD* represent *R. sanguineum* var. *sanguineum*, *R.* var. *haemaleum*, and *R.* var. *didymoides*, respectively. qRT-PCR expression results of the anthocyanin genes of the three varieties given as bar charts and shown as means of three biological replicates with standard errors. Analyses were performed on the flower bud samples. Relative mRNA (y-axis) expression levels were normalized to *GAPDH* (FN552706)

As shown in Fig. 4, the results showed that the vast majority of genes in anthocyanin synthesis, modification and transfer enzymes were more highly expressed in *RsH*, over *RsD* and *RsS*, and genes assigned to hydrolytic enzymes were down-regulated in *RsH*. There were nine genes assigned to six enzymes shared in two taxa comparisons with divergent reflectance spectra (*RsH* vs. *RsD* and *RsH* vs. *RsS*), including *RsBGLU1/3*, *RsPER1/3/4*, *RsCHI1*, *RsFLS1*, *RsANS2* and *RsAT2* (Fig. 3d; Figure S7, S8). However, only a handful of DEGs, including *Rs4CL*, *RsBGLU7* and *RsPER1* exhibited higher expression in *RsS* over *RsD*. Moreover, in *RsD*, *RsBGLU4/5*, *RsPER5/7/8*, *RsCAD1/2*, *RsAT1*, *RsOMT* were higher expressed than in *RsS* (Fig. 3d; Fig. 4; Figure S9). A tentative schematic flowchart hypothesizing the anthocyanin biosynthesis pathway relevant to the flower coloration in the *R. sanguineum* complex is proposed in Fig. 4.

### Sequence diversity in promoter regions of genes involved in the anthocyanin pathway

The promoter sequences (2 kb upstream of the translation initiation sites) of each gene showed a high conservation in both sequence similarity (between 98.6 to 99.7%) and GC contents, though they differed at certain positions (Table S8; Figure S10). A detailed analysis of *cis-*acting elements in these promoters with a focus on MYB transcription binding sites revealed that, with the exception of *RsANS* and *RsF3′H*, all other 11 candidate genes featured variation in at least one *cis-*motif (Figure S10). These promoters also had variations in other types of *cis-*motifs (Table S8). These data thus provide a plausible link between promoter regions (variations in sequences and *cis*-acting elements) and the differential expression of the respective anthocyanin genes.

## Discussion

The flavonoid / anthocyanin metabolism is thought to be one of the most important pathways that contributes to flower pigmentation and is catalyzed by a multi-enzyme complex [10]. The genetic basis underlying the flower coloration has recently been studied at species level in *Erica*, a closely related genus to *Rhododendron* in Ericaceae [35, 36]. These studies demonstrated that losses of expression of single pathway genes, or collapse of a pathway due to loss of transcription factor or loss of function mutations underlie color changes [35]. While the color changes in these studies concerned variation between species, in this study, we endeavored to elucidate the genetics of color change between more closely related evolutionary entities, between varieties of the *R. sanguineum* complex. These changes may be more subtle as the evolutionary distance is smaller than between species and maybe less affected by homoplasies and reflect more accurate evolutionary pathway changes. One of the drawbacks of the previous studies was the lack of transcriptome data that did not allow the detection of gene paralogs [35, 36]. Indeed, in the present study we found many anthocyanin genes to be part of small gene families (Fig. 3d), and therefore more precise in unravelling the anthocyanin pathway.

By conducting comparative transcriptome and confirmative qRT-PCR experiments, we found similar patterns of differentially expressed genes in pairwise comparisons of taxa characterized by similar reflectance spectrum differences, i.e. *RsH* vs. *RsD* and *RsH* vs. *RsS*. Consequently, a lower number of DEGs were found when comparing the two taxa with a more similar reflectance spectrum, *RsS* vs. *RsD*. Thus, the magnitude of phenotypic differences (i.e. differences in anthocyanin content) between closely related taxa may be the result of respective color gene expression level changes in flower tissues. Here, we identified nine genes assigned to six enzymes shared in the two taxa comparisons, *RsH* vs. *RsD* and *RsH* vs. *RsS* (Figure S7, S8). In our result of the reflectance spectrum, the brightness parameter (B) (often refers to intensity) was lowest and the ratio of saturation (S) and hue (H) highest in variety *RsH* (Fig. 2c, d), which indicated a high anthocyanin content [29, 31]. Likewise, previous studies stated that flower color intensity is thought to be largely determined by the amount of anthocyanin [44, 45], i.e., as the anthocyanin content increases the flower color deepens.

High expression levels were shown to intensify colors [46] and we found different genes that may have contributed to the deep blackish crimson colored flowers of *R.* var*. haemalum* (*RsH*). Our results showed that *RsCHI1*, *RsFLS1*, *RsANS2* and *RsAT2* that were highly expressed in taxa comparisons involving *RsH* were significantly expressed in this variety corresponding to its deep blackish crimson flowers (Fig. 3d; Fig. 4). This finding is similar to the case of fruit development of blueberries, where a correlation between expression of pathway genes and anthocyanin production was found [46]. In the flavonoid / anthocyanin pathway, *CHI*, highly expressed only in *RsH*, was demonstrated to convert chalcone to flavanone. The *ANS* gene, also highly expressed only in *RsH*, played a key role in catalyzing the synthesis of colorless leucoanthocyanidins into colored anthocyanidins. For example, Zhang et al. [47] reported that *CHI* is expressed at significantly higher levels in red leaves than in paler colored ones in lettuce (*Lactuca sativa*). Zhao et al. [48] found that the lower expression of *ANS* of *Paeonia lactiflora* resulted in lower anthocyanin accumulation and resulted in yellow colors. In our study, *RsANS* might be the reason for the weak anthocyanin accumulation in *RsD*, and high accumulation in *RsH* to achieve the relative high color intensity petals in the latter. Generally, the structure of anthocyanidin pigments accumulated by *ANS* catalysis are unstable and are stabilized by subsequent acylation by anthocyanin acyltransferase (*AT*) to form stable anthocyanins [10]. It was reported that when *AT* was inhibited, the petals became pale [49]. This is shown in our results where the *RsAT* gene was highly expressed in *RsH* with low brightness (high flower color intensity), and lower for the other two varieties that have paler corollas. Intriguingly, the *FLS* gene was highly expressed in *RsH*. *FLS* competes for the same substrate as *DFR*, resulting in the production of different flavonoids and anthocyanins [10, 49], indicating co-pigmentation effects [50]. Color changes due to co-pigmentation increases the color intensity of flowers [51]. In our results, co-pigmentation, normally with flavonols contributed by *RsFLS*, may have resulted in intensely colored anthocyanins that shift color toward deep blackish crimson probably. This is in line with the study of the reddish-purple color in the petals of *R. simsii* flowers [52].

In the comparison of *RsS* vs. *RsD* with red and yellow flushed pink corolla tubes respectively, the comparative transcriptome analysis suggested only 12 paralogs of six genes related to flower color biosynthesis to be differentially expressed (Figure S9). It is likely that the relatively smaller difference in the reflectance spectrum (i.e. flower color difference) between *RsS* and *RsD*, which is the reason for the lower number of DEGs related to anthocyanin biosynthesis. Three of the six genes highly expressed in *RsD*, i.e. *RsBGLU*, *RsCAD*, and *RsPER*, belong to catabolic enzymes and thus maybe responsible for the lighter color. Our findings were consistent with previous studies such as, Yang et al. [53] who investigated flower color change of two tree peony cultivars and their results indicated a sharp decrease in anthocyanins to be responsible for the change in color from red to orange and yellow. Similarly, Zhu et al. [54] proposed that differential expression of *NnOMTs* may be related to petal color differences in two *Nelumbo nucifera* cultivars with yellow and white flowers. But these might be different from the findings of Le Maitre et al. [35, 36] for *Erica* species with yellow flowers. For these, they found either normal gene expression or absence of expression of *F3*′*H* and absence of a MYB recognition element. Other pigments, such as carotenoids could also influence the color of flowers [10]. However, these have seldom be detected in species of *Rhododendron* [11, 33]. Other genes, such as *RsCHS*, *RsF3H*, *RsF3*′*H* and *RsGST* were found also highly expressed in *RsH* when compared to either *RsD* or *RsS*. These are single copy genes and likely candidates to explain the dark flower color in this variety.

Our results further showed that the expression of *RsBGLU1/3*, *RsPER1/3/4* genes associated with metabolic enzymes were significantly higher in *RsD* and somewhat higher in *RsS* compared to *RsH*. In the flavonoid / anthocyanin pathway (Fig. 4), the two genes were assigned to the “early biosynthesis genes” (EBGs) related to the early phenylpropanoid biosynthesis pathway. They were shown to be involved in the hydrolysis of cinnamic acid and P-coumaroyl-CoA to coumarine and lignin respectively [13]. *β*-glucosidases (*BGLU*) and peroxidases (*PER*) have been shown to be responsible for anthocyanin degradation in many plants [24, 25]. In general, anthocyanin accumulation is determined by the balance of biosynthesis and degradation. In our study, the varieties *RsD* and *RsS* may have reduced anthocyanin accumulation because of the catabolism of early substrates [24] and degradation of mature anthocyanins in the flavonoid / anthocyanin pathway, thus resulting in low flower color intensity. It may well be that the floral coloration among these varieties were associated with flux shifts (anthocyanin contents) through the pathway, leading to the differences in color intensity.

However, all candidate genes mentioned above are classified as structural genes. MBW (MYB-bHLH-WD40) protein complex genes were not significantly differently expressed. One possible explanation might point to the sampling approach here, using only one stage (late flower bud stage). Yang et al. [19] demonstrated that MBW transcription factors collectively regulate anthocyanin accumulation at the transcriptional level, particularly at the initial stages of flower coloration, and may not be present in later flower bud stages. In addition, the MBW complex can bind to the promoter of anthocyanin genes, and the MYB binding site of promoter sequences plays a vital role in anthocyanin synthesis [55]. In the present study, the promoter sequences of each genes was conserved across the three varieties in general, but featured variation in *cis*-motifs such as MYB binding site in 11 genes, except for *RsANS* and *RsF3*′*H* (Table S8; Figure S10). Our results may also, on some level, give a support to an *evo-devo* hypothesis [56] where phenotypic differences, particular for closely related species, are more likely to be triggered by *cis*-regulatory regions than the protein-coding regions of genes, but they are thought to be relatively free of negative pleiotropic effects on fitness, while mutational changes to transcription factor genes are the least likely due to potentially more wide-reaching effects [35]. Furthermore, some studies revealed that methylation levels in the promoter regions of *MdGST* [18] and *OgCHS* [57] are linked significantly with gene expression levels and thus anthocynin diversity. However, the GC content in the promoter regions of each gene featured a similar level among varieties (Figure S10), suggesting that DNA methylation is unlikely the reason for color diversity in the *R. sanguineum* complex. Thus, we suggest that the regulation and transcription of anthocyanin pathway genes may not be independent processes, although they are expressed separately, they are collectively contributing to anthocyanin accumulation [55, 58]. Nevertheless, we obtained initial findings on the flavonoid / anthocyanin pathway in the *R. sanguineum* complex, but additional experiments are needed to further investigate the contribution of each genetic component in this scenario.

It has been proposed that evolutionary transitions in flower color are often attributed to pollinator-mediated selection, which may have contributed to a niche occupation and consequent reproductive isolation and diversification / speciation [5]. For example, bees have three types of photoreceptors peaking in the UV, blue and green range of the spectrum corresponding to 344, 438 and 560 nm, respectively [59], whereas birds are tetrachromatic and have further receptors sensitive to red light at 600 to 620 nm [60].

In some pollination studies of *Rhododendron*, birds, bees, butterflies and sphingid moths were found to represent the predominant pollinators for *Rhododendron* species [40–42]. Song et al. [61] proposed that sunbird and bumblebee were potential pollinators for *R*. *delavayi* and *R*. *edgeworthii* with red-flowered and white-flowered, respectively, while Epps et al. [62] observed pollination by butterfly in the yellow-orange flowered of *R. calendulaceum*. This indicates that pollinators show flower color preferences, and flowers with similar colors tend to attract specific pollinators, even between conspecific populations or among closely related species [6, 59]. Consequently, spatiotemporal fluctuations in pollinator assemblages could lead to a shift in flower color and vice versa [5]. However, in some closely related sympatric taxa, incomplete reproductive barriers contribute to hybridization and play critical roles in gene flow [63]. Hybridization in low reproductive isolation can pass around color genes frequently, especially in a limited gene pool, as indicated here for the varieties of the *R. sanguineum* complex as they do not form monophyletic groups (Figure S3). Our results are in agreement with reports showing important changes in flower color as a result of relatively simple genetic changes [64]. We have inferred these in the closely related varieties here. This situation may initiate a shift in pollinator assembly and reproductive isolation consequently be reinforced over time. Our study may thus demonstrate an incipient sympatric speciation pattern induced by flower color differentiation, as has been observed for birds by behavioral isolation [65], and in plant species by geology-edaphic divergence [66], or geographic variation [67].

## Conclusions

In this study, Illumina transcriptome sequencing (RNA-seq) and genome resequencing were applied to analyze changes in flower color gene expression of field-collected samples of three varieties of the *R. sanguineum* complex. This study provided preliminary insights into genetic mechanisms underlying the flower color divergence in the *R. sanguineum* complex. Our results indicated that variation in the flower color of the varieties are linked to differences in expression levels and to some extent to *cis-*acting regulation of anthocyanin biosynthesis genes and anthocyanin degradation genes, rather than loss of function mutations. The deep blackish crimson flowered *R*. var. *haemalum* showed a high expression for almost all anthocyanin genes, while the bright crimson flowered *R*. var. *sanguineum* had high expression levels for genes in the initial steps of anthocyanin synthesis and the yellow flushed pink flowered *R*. var. *didymoides* had low or medium expression levels for most anthocyanin genes. The latter also had high expression levels for anthocyanin degradation genes that may added to the low anthocyanin contents of its flowers. The findings differ from a previous study on *Erica* species (also Ericaceae) where frameshift mutations in anthocyanin genes and MYB recognition elements were found to be responsible for red-yellow color shifts. Some hypotheses can be put forward to explain the flower color variation in the complex: the varieties are too closely related to have acquired mutations in coding regions of anthocyanin genes or transcription factors, and their color variations due to differences in expression levels that can be induced by *cis*-acting regulation in promoters, and the balance between genes in the anabolism and catabolism anthocyanin pathway. This complex has at present a complicated relationship involving hybridization and gene flow among the varieties, but if reinforcement by pollinator preferences develop further, may develop into a case of pollinator-driven incipient sympatric speciation.

## Methods

### Samples collection

Samples of three varieties of the *R. sanguineum* complex (Fig. 1), namely *R. sanguineum* var. *sanguineum* (*RsS*, with bright crimson flowers), *R.* var. *haemaleum* (*RsH*, with deep blackish crimson flowers), *R.* var. *didymoides* (*RsD*, with yellow flushed pink flowers), co-existing in the Gaoligong Mountains (N 27°47′11.40″, E 98°27′35.28″) which located in northwest Yunnan, China, were collected in June 2018. Flower tissues at the late bud stage (Fig. 1b) and leaf buds (Fig. 1c) from three individuals per variety were sampled, immediately frozen with liquid nitrogen. Leaf tissues from the same individuals were sampled and dried with silica gel at the same time, as were flowers for corolla reflectance spectra measurements. The nine individuals were sampled across a small range (20 m^2^) under similar climatic conditions and environmental factors, such as soil, temperature, precipitation and sunlight radiation. Vouchers of each individual were collected and deposited at the Herbarium of Kunming Institute of Botany (KUN), Chinese Academy of Sciences.

### Flower reflectance spectra measurements

Absorption of light in the visible spectrum by plant pigments produces a unique spectral reflectance signature. To obtain the reflectance spectra of the different flower colors of the three varieties, a spectrometer approach was used that quantified anthocyanin corolla pigments [11, 68]. Five fresh, healthy and fully opened flowers of each sampled plant were measured (15 per variety, 45 samples in total) in the field. Two independent spectra readings were taken from the same position in the flower, half way down the corolla tube. Diffuse reflectance spectra were measured in the range of 200–800 nm using an USB2000+ spectrometer with a deuterium / tungsten halogen light source (Ocean Optics, Dunedin, FL, USA) with a 3 s integration time and boxcar of 12. True black and true white control references were scanned before each sample measurement. Color data were processed using Optic 2009 SpectraSuite (Ocean Optics) software. Spectra were truncated to 300–700 nm and averaged per measurement per variety using the *pavo* package in *R* [69, 70]. The ratio between saturation and hue (S/H) was used to determine the anthocyanin content of the flowers [31].

### RNA and DNA extraction and Illumina sequencing

Total RNA was extracted and purified separately from flower bud and leaf bud tissues using a Spectrum TM Plant Total RNA Kit (STRN250, Sigma) according to the manufacturer’s protocols. Three biological replicates (from three plants) for each flower bud and leaf bud material were included for each variety. Genomic DNA was extracted from leaves using a DNeasy Plant kit (QIAGEN). The RNA and DNA quality and quantity were assessed with a NanoDrop 2000 spectrophotometer (Thermo Fisher Scientific, Waltham, MA, USA). Transcriptomic and genomic libraries were generated according to the manufacturer’s protocol using the NEBNext Ultra™ RNA/DNA Library Prep Kit (NEB, MA, USA) and sequenced on an Illumina HiSeq X Ten sequencing platform (San Diego, CA, USA), generating approximate 6 Gb and 7 Gb (~ 10x) paired-end reads (2 × 150 bp) of each sample for RNA and DNA libraries, respectively. Library preparation and Illumina sequencing were performed at Novogene Bioinformatics Technology Co., Ltd. (Beijing, China).

### Data processing, de novo assembly and mapping

The raw data were first filtered by removing reads with adapter sequences, reads containing poly-Ns, reads with ambiguous nucleotides and those of low-quality, then the phred scores (Q20, Q30) were calculated using SOAPnuke [71]. The quality of the remaining reads was evaluated with FastQC, including GC-content, sequence length distribution and sequence duplication level of the clean data [72]. All subsequent analyses were based on the clean data.

To obtain a reference-level transcriptome assembly, the cleaned reads from each variety (leaf buds, flower buds, in three replicates) were combined and were assembled de novo with Trinity v. 2.6.5 [73]. All Trinity parameters were set to default except the minimum kmer coverage (set to 2) and minimum contig length (set to 200). Assembly statistics were obtained using the TrinityStats.pl script in the Trinity package. We also used HISAT2 v. 2.1.0 [74] to assess assembly quality, by mapping reads back to the assembled transcripts to count the overall alignment rates.

### Assembly filtering and assessment of completeness

All assembled transcripts were filtered to reduce the redundancy and complexity as follows: first, we used CD-HIT-EST v. 4.7.0 [75], with setting word length to 10 and sequence identity threshold to 0.95, to remove duplicates. Then, Corset v. 1.07 [76] was used to cluster the transcript sequences and filter out redundant transcripts to extract one representative transcript per gene. In this case, we only kept the longest transcript per gene. Downstream analyses were performed on the final filtered transcripts. To determine the transcriptome completeness of each assembly, Benchmarking Universal Single-Copy Orthologs tools (BUSCO, v. 4.0.6) [77] was used to obtain the percentage of single-copy orthologs represented in the embryophyte database and also to evaluate the completeness of transcript assemblies.

### Ortholog prediction and functional annotation

Open reading frames (ORFs) were predicted from each filtered assembled transcripts using TransDecoder v. 5.5.0 [73]. This pipeline included principally two steps. In the first step, the longest ORF per transcript was predicted with a cut-off minimum length of 100 amino acids. Then, the predicted ORFs were scanned to find homology profiles using BLASTP v. 2.5.0 searches with a cut-off e-value of 1e-10 against a curated protein database for *R. delavayi*, a species closely related to *R. sanguineum*, downloaded from the whole genome sequencing project deposited in GigaDB [78]. All best-hit coding peptides were retained for the final prediction of the amino acid sequence. When there was more than one prediction within a transcript, we selected the top scoring ORF for each transcript. We used CD-HIT v. 4.7.0 [75] to further reduce redundancy of the final predicted amino acids with the sequence identity threshold setting of 0.95. Orthologous clusters (orthogroups) of protein sequences amongst the three varieties were identified with OrthoVenn2 [43], a web server platform, using the e-value threshold of 1e-10. The one-to-one single copy orthologs among the annotated ORF datasets of the three varieties were used for subsequent analyses. Orthologous protein sequences of *R.* var. *didymoides* were used as proxies for searching against protein databases, including NCBI non-redundant (NR) and UniProtKB/Swiss-Prot with BLASTP v. 2.5.0, setting the e-value cutoff to 1e-10. We also performed additional functional annotations with DIAMOND [79] hits against eggNOG database [80], which summarized available functional information from the different proteins databases, including GO, COGs/KOGs, and KEGG. The best hit was used as final annotation.

### Read mapping, SNP calling and clustering

For each variety, clean reads (of flower bud and leaf bud combined) for each of the three replicate plants were aligned separately to the reference transcriptome (Orthologous clusters) using HISAT2 v. 2.1.0 [74] with default parameter settings. SAMtools v. 1.9 (https://github.com/samtools/), and Picard tools v. 2.21.8 (http://broadinstitute.github.io/picard/) were used to sort, mark and remove duplicated reads, and reorder the bam alignment results for each of the nine samples. We used GATK v. 4.1.5 [81] to perform SNP calling and Plink v. 1.9 (http://pngu.mgh.harvard.edu/purcell/plink/) to filter the SNPs with the parameters settings --*geno* 0.1 --*maf* 0.01 --*indep-pairwise* 50, 10, 0.2. Finally, the qualified SNPs were combined into a single VCF file which was used as input into SNPhylo v. 20180901 [82] to reconstruct the phylogenetic relationships among the samples using maximum likelihood, with 1000 bootstrap replicates for branch support. To further investigate the distribution of genetic variation, a principal component analysis (PCA) was conducted on the SNP variation in *R* [69].

### Transcript abundance and differential expression analyses

Gene expression levels (abundance estimation) were calculated by mapping all of the paired-end reads from the flower bud samples separately for each biological replicate back to the one-to-one single copy orthologs of the reference transcriptome for each variety using RSEM v. 1.3.1 [83], and Bowtie2 v. 2.3.5 was used for alignment [84]. Because each variety assembly was generated independently de novo without reference, Trinity ID headers were assigned to each variety randomly. To ensure each quantification file was assigned the same ID header and thus could be integrated, we replaced the transcript ID generated by Trinity, with its respective single copy ortholog name (orthologous cluster). After obtaining the gene expression abundance for each biological replicate flower bud sample, we generated a gene expression matrix based on the fragments per kilobase of exon per million fragments mapped reads (FPKM). A read count matrix generated with a Trinity script was then used for differentially expressed gene (DEGs) analyses. The differential analysis was performed with DESeq2 package [85] in *R* among the three varieties to identify the DEGs by pairwise comparisons. DEGs were considered those with false discovery rate (FDR) adjusted *p* values ≤0.05 and absolute values of log2 (fold change) ≥ 1. To compare gene expression values across the three varieties, we used the trimmed mean of M-values normalization (TMM), as implemented in the *R* package edgeR [86]. All downstream analyses were implemented based on the normalized expression data matrix (TMM normalization). The DEGs from each comparison among the varieties were selected for further functional enrichment analysis. Based on the functional annotation of all orthologous genes, all annotation terms were extracted by in-house Perl scripts and imported into an *R* package AnnotationForge to generate an OrgDB organism annotation object, which contained mappings correspondingly between gene ID and other identifiers in the databases as described above. The GO (Gene Ontology) and KEGG (Kyoto Encyclopedia of Genes and Genomes) enrichment analysis of differentially expressed genes (DEGs) were implemented by the clusterProfiler package [87] in *R*.

### Statistical analyses of the expression profiles

All expression analyses were performed separately for the three biological replicates in each variety. We constructed a gene expression matrix with nine columns and 15,164 lines. Each column represented a sample and each line corresponded to the expression of an orthologous gene. The data matrix was used to calculate the Pearson’s correlation coefficient (*r*) between all pairs of samples. The symmetrical heat map and principal component analysis (PCA) of all samples were carried out with the *R* package Pheatmap v. 1.0.12 (https://CRAN.R-project.org/package=pheatmap) and PCAtools v. 1.1.0 (https://github.com/kevinblighe/PCAtools), respectively. GO terms and metabolic pathways with *p* values ≤0.05 were considered significantly enriched by DEGs.

### Validation by quantitative real-time PCR (qRT-PCR)

To verify the reliability of the RNA-seq results with respect to the anthocyanin biosynthesis pathway, 13 genes were selected for qRT-PCR analysis. First strand cDNA libraries were synthesized using a BioRT Master HiSensi cDNA First Stand Synthesis Kit (Bioer, Hangzhou, China) and diluted 20-fold as templates. QRT-PCR was performed with three biological replicates (plants) using BioEasy master mix SYBR Green (Bioer, Hangzhou, China) on a QuantStudio™ 7 Flex Real-Time PCR System (Applied Biosystems, CA, US). The qRT-PCR amplification conditions were as follows: denaturation at 95 °C for 1 min, followed by 40 cycles of denaturation at 95 °C for 15 s, annealing and extension together at 60 °C for 60s. The primers for the 12 genes are listed in Table S7. *GLYCERALDEHYDE-3-PHOSPHATE DEHYDROGENASE* (*GAPDH*) of *R. simsii* (GenBank acc. no. FN552706) was used as an internal control for normalization [50], and relative expression levels were estimated using the 2^−ΔΔCT^ method [88].

### Identification and characterization of promoter sequence diversity

To further understand whether the expression levels were affected by *cis*-regulatory elements and DNA methylation of promoters in these anthocyanin related genes, the cDNA sequences for the 13 DEGs were searched against the published *Rhododendron* genome [78] with BLASTn and the best hit gene with highest sequence similiarity was treated as the corresponding reference. We then mapped the clean reads of each genomic sequencing variety (three individuals combined) to each of the *Rhododendron* reference sequences and built a consensus sequence by sam2consensus.py script (https://github.com/edgardomortiz/sam2consensus). The resulting consensus sequences (fasta file) of each variety for each gene were generated. Each 2 kb promoter sequence upstream of the translation start site was shorten from the consensus sequences and *cis*-acting regulatory elements were then predicted by PlantCARE (http://bioinformatics.psb.ugent.be/webtools/plantcare/html/) databases. TBtools v. 1.068 [89] was then used to visualize the distribution of *cis*-acting elements. Pairwise alignment of promoter sequences of each gene from the three varieties to each reference sequence using a online platform, mVISTA [90] and basepair identity was graphed in a sliding window of 50 bp in a range of 75 to 100%. The GC content of promoters of each gene across the varieties was calculated as well with a sliding window of 50 bp, and subsequently visualized by ggplot2 package in *R* [69].

## Supplementary Information

**Additional file 1: Table S1.** Statistics of the sequencing, assembly, and filtering of transcriptomes of samples of three varieties of the *Rhododendron sanguineum* complex. **Table S2.** Basic information of the transcriptome sequencing and alignment rates of 18 RNA libraries of two tissues of three varieties of the *Rhododendron sanguineum* complex. **Table S3.** BUSCO statistics for transcriptome assembly quality assessment of three varieties of the *Rhododendron sanguineum* complex. **Table S4.** Orthologous cluster statistics across three varieties of the *Rhododendron sanguineum* complex. **Table S5.** Identification and annotation of orthologous clusters across three varieties of the *Rhododendron sanguineum* complex. **Table S6.** List of the 40 predicted anthocyanin-associated genes and paralogs found in the *Rhododendron sanguineum* complex. **Table S7.** Primer information used for qRT-PCR validation of 12 gene paralogs involved in anthocyanin synthesis in three varieties of the *Rhododendron sanguineum* complex. **Table S8.** Statistical information of promoters for all genes among the *Rhododendron sanguineum* complex. **Figure S1.** BUSCO quality assessment results of transcriptome assemblies for three varieties of the *Rhododendron sanguineum* complex (a) and annotation of 15,164 orthologous cluster hits against six different databases (b). RsS – *R. sanguineum* var. *sanguineum*; RsH – *R. sanguineum* var. *haemaleum*; RsD – *R. sanguineum* var. *didymoides*. **Figure S2.** Annotation of the 15,164 one to one single copy orthologs among three varieties of the *Rhododendron sanguineum* complex through interrogation against three different databases. **Figure S3.** Phylogenetic and genetic clustering results for the three varieties of the *Rhododendron sanguineum* complex. **Figure S4.** Gene expression patterns of all 18 samples of three varieties of the *Rhododendron sanguineum* complex. **Figure S5.** Volcano plots of differentially expressed genes (DEGs) based on pairwise comparisons of the three varieties of the *Rhododendron sanguineum* complex. **Figure S6.** Heatmap and results of the functional enrichment of differentially expressed genes (DEGs) among the three varieties of the *Rhododendron sanguineum* complex. **Figure S7.** Hierarchical clustering of normalized expression levels of 23 candidate genes show distinct gene expression profiles in comparison of *RsH* and *RsD*. Variety code as in Figure S1. Red indicates high expression, and blue indicates low expression. **Figure S8.** Hierarchical clustering of normalized expression levels of 25 candidate genes show distinct gene expression profiles in comparison of *RsH* and *RsS*. Variety code as in Figure S1. Red represents high expression, and blue represents low expression. **Figure S9.** Hierarchical clustering of normalized expression levels of 12 candidate genes show distinct gene expression profiles in comparison of *RsS* and *RsD*. Variety code as in Figure S1. Red represents high expression, and blue represents low expression. **Figure S10.** A common layout of the promoter architecture of the 13 anthocyanin genes across varieties. Variety code as in Figure S1.

## Data Availability

The raw data can be accessed from the NCBI Sequence Read Archive (SRA) platform under the accession number PRJNA720238 (http://www.ncbi.nlm.nih.gov/bioproject/720238).

## Abbreviations

4CL: 4-coumarate-CoA ligase
ANR: Anthocyanidin reductase;
ANS: Anthocyanin synthase
AT: Acetyltransferase
BGLU: Beta-glucosidase
C4H: Cinnamic acid4-hydroxylase
CAD: Cinnamyl alcohol dehydrogenase
CHI: Chalcone isomerase
CHS: Chalcone synthase
DFR: Dihydroflavonol-4-reductase
F3′H: Flavanone 3′-hydroxylase
F3H: Flavanone 3-hydroxylase
FLS: Flavonol synthase
GST: Glutathione s-transferase
MBW: MYB-bHLH-WD40 complex
OMT: O-methyltransferase
PAL: Phenylalanine ammonia-lyase
PER: Peroxidase
GAPDH: Glyceraldehyde 3-phosphate dehydrogenase
RNA-seq: RNA sequencing
DEGs: Differentially expressed genes
BLAST: Basic local alignment search tool
BUSCO: Benchmarking universal single-copy orthologs
NR: NCBI non-redundant
UniProtKB: Universal protein knowledgebase
COGs: Clusters of orthologous groups
eggNOG: Evolutionary genealogy of genes: non-supervised orthologous groups
GATK: Genome analysis toolkit
GO: Gene ontology
KOG: Eukaryotic orthologous groups of proteins
KEGG: Kyoto encyclopedia of genes and genomes
FDR: False discovery rate
FPKM: Fragments per kb per million fragments
PCA: Principal component analysis
SNP: Single nucleotide polymorphism
TMM: Trimmed mean of m-values.
