# Supplementary material for: Differential expressions of anthocyanin synthesis genes underlie flower color divergence in a sympatric Rhododendron sanguineum complex
Source: BMC Plant Biol. 2021 Apr 28;21:204. doi: 10.1186/s12870-021-02977-9 (PMC8082929; doi:10.1186/s12870-021-02977-9)
Supplement: Supplementary file 1 — Additional file 1: Table S1. Statistics of the sequencing, assembly, and filtering of transcriptomes of samples of three varieties of the Rhododendron sanguineum complex. Table S2. Basic information of the transcriptome sequencing and alignment rates of 18 RNA libraries of two tissues of three varieties of the Rhododendron sanguineum complex. Table S3. BUSCO statistics for transcriptome assembly quality assessment of three varieties of the Rhododendron sanguineum complex. Table S4. Orthologous cluster statistics across three varieties of the Rhododendron sanguineum complex. Table S5. Identification and annotation of orthologous clusters across three varieties of the Rhododendron sanguineum complex. Table S6. List of the 40 predicted anthocyanin-associated genes and paralogs found in the Rhododendron sanguineum complex. Table S7. Primer information used for qRT-PCR validation of 12 gene paralogs involved in anthocyanin synthesis in three varieties of the Rhododendron sanguineum complex. Table S8. Statistical information of promoters for all genes among the Rhododendron sanguineum complex. Figure S1. BUSCO quality assessment results of transcriptome assemblies for three varieties of the Rhododendron sanguineum complex (a) and annotation of 15,164 orthologous cluster hits against six different databases (b). RsS – R. sanguineum var. sanguineum; RsH – R. sanguineum var. haemaleum; RsD – R. sanguineum var. didymoides. Figure S2. Annotation of the 15,164 one to one single copy orthologs among three varieties of the Rhododendron sanguineum complex through interrogation against three different databases. Figure S3. Phylogenetic and genetic clustering results for the three varieties of the Rhododendron sanguineum complex. Figure S4. Gene expression patterns of all 18 samples of three varieties of the Rhododendron sanguineum complex. Figure S5. Volcano plots of differentially expressed genes (DEGs) based on pairwise comparisons of the three varieties of the Rhododendron sangu [file 12870_2021_2977_MOESM1_ESM.pdf]

Differential expressions of anthocyanin synthesis genes underlie flower color divergence in a sympatric *Rhododendron sanguineum* complex

Lin-Jiang Ye[1,2,3,4], Michael Möller[5], Ya-Huang Luo[1], Jia-Yun Zou[1,4], Wei Zheng[1,4], Yue-Hua Wang[3], Jie Liu[1], An-Dan Zhu[2], Jin-Yong Hu[1], De-Zhu Li[2,4*], Lian-Ming Gao[1,6*]

[1] CAS Key Laboratory for Plant Diversity and Biogeography of East Asia, Kunming Institute of Botany, Chinese Academy of Sciences, Kunming, Yunnan 650201, China;

[2] Germplasm Bank of Wild Species, Kunming Institute of Botany, Chinese Academy of Sciences, Kunming, Yunnan 650201, China;

[3] School of Life Sciences, Yunnan University, Kunming, Yunnan 650091, China;

[4] University of Chinese Academy of Sciences, Beijing 10049, China;

[5] Royal Botanic Garden Edinburgh, Edinburgh EH3 5LR, United Kingdom;

[6] Yunnan Lijiang Forest Ecosystem National Observation and Research Station, Kunming Institute of Botany, Chinese Academy of Sciences, Lijiang 674100, Yunnan, China.

Supplementary Information

Table S1 Statistics of the sequencing, assembly, and filtering of transcriptomes of samples of three varieties of the *Rhododendron sanguineum* complex.

	R. var. *sanguineum*	*R*. var. *haemaleum*	*R*. var. *didymoides*
Statistics for cleaned reads			
F_01	36,571,024	37,231,394	37,988,360
F_02	35,787,304	35,754,758	42,143,954
F_03	32,518,132	33,982,970	43,353,842
L_01	42,576,920	37,766,022	41,101,370
L_02	32,111,674	33,875,762	39,540,704
L_03	42,334,614	40,560,244	42,437,260
total	221,899,668	219,171,150	246,565,490
Statistics for assemblies			
***De* novo**			
No._seqs	208,830	279,118	188,413
No._bases	174,217,618	188,199,415	149,084,170
Mean_len.	834.26	674.26	791.26
N50 (bp)	1,474	1,226	1,412
**CD-HIT_filt**			
No._seqs	169,104	237,035	155,126
No._bases	121,720,352	140,062,299	107,665,078
Mean_len.	719.8	590.89	694.05
N50 (bp)	1,226	965	1,194
**CORSET_filt**			
No._seqs	137,335	171,725	117,976
No._bases	98,620,485	104,976,701	81,809,051
Mean_len.	718.1	611.31	693.44
N50 (bp)	1,125	903	1,068

Note: No._seqs – number of sequence reads; No._bases – number of nucleotide bases; Mean_len. – mean length of transcripts; N50 – length of the smallest contig in the set that contains the fewest (largest) contigs whose combined length represents at least 50% of the assembly.

Table S2 Basic information of the transcriptome sequencing and alignment rates of 18 RNA libraries of two tissues of three varieties of the *Rhododendron sanguineum* complex.

sample name	clean reads	clean bases (bp)	Q20	Q30	GC content	mapping rate
F_RsS_01	36,571,024	5,485,653,600	98.11%	95.05%	48.54%	92.57%
F_RsS_02	35,787,304	5,368,095,600	97.65%	93.58%	47.67%	91.58%
F_RsS_03	32,518,132	4,877,719,800	97.60%	93.48%	48.02%	93.26%
F_RsH_01	37,231,394	5,584,709,100	98.07%	94.87%	47.02%	90.86%

F_RsH_02	35,754,758	5,363,213,700	97.89%	94.65%	47.28%	84.25%
F_RsH_03	33,982,970	5,097,445,500	97.95%	94.50%	47.81%	88.25%
F_RsD_01	37,988,360	5,698,254,000	98.05%	94.81%	47.22%	93.21%
F_RsD_02	42,143,954	6,321,593,100	98.04%	94.86%	49.53%	94.85%
F_RsD_03	43,353,842	6,503,076,300	96.75%	91.87%	48.21%	93.76%
L_RsS_01	42,576,920	6,386,538,000	97.78%	93.87%	47.86%	94.20%
L_RsS_02	32,111,674	4,816,751,100	97.81%	93.92%	47.14%	92.72%
L_RsS_03	42,334,614	6,350,192,100	98.34%	95.35%	48.01%	92.59%
L_RsH_01	37,766,022	5,664,903,300	98.24%	95.09%	47.76%	93.50%
L_RsH_02	33,875,762	5,081,364,300	98.27%	95.16%	48.04%	92.20%
L_RsH_03	40,560,244	6,084,036,600	98.05%	94.59%	47.42%	92.74%
L_RsD_01	41,101,370	6,165,205,500	98.09%	94.74%	47.79%	94.00%
L_RsD_02	39,540,704	5,931,105,600	97.81%	94.25%	48.20%	92.69%
L_RsD_03	42,437,260	6,365,589,000	98.01%	94.52%	47.84%	94.08%

Note: F – RNA-seq libraries from late floral bud tissue; L – RNA-seq libraries from leaf bud tissue. RsD – *R. sanguineum* var. *didymoides*; RsH – *R. sanguineum* var. *haemaleum*; RsS – *R. sanguineum* var. *sanguineum*; Q20 – phred score that the base call accuracy of 99%; Q30 – phred score that the base call accuracy of 99.9%; 01-03 – biological replicates.

Table S3 BUSCO statistics for transcriptome assembly quality assessment of three varieties of the *Rhododendron sanguineum* complex.

	R. var. *sanguineum*	*R.* var. *haemaleum*	*R.* var. *didymoides*
Complete BUSCOs	1,487 (92.1%)	1,502 (93.1%)	1,484 (91.9%)
Fragmented BUSCOs	53 (3.3%)	46 (2.9%)	54 (3.3%)
Missing BUSCOs	74 (4.6%)	66 (4.0%)	76 (4.8%)
Total BUSCOs groups searched	1,614 (100%)	1,614 (100%)	1,614 (100%)

Note: complete BUSCOs include single-copy and duplicate BUSCOs. Percentages of the total number of BUSCO groups searched are shown in parentheses.

Table S4 Orthologous cluster statistics across three varieties of the *Rhododendron sanguineum* complex.

Taxon	Proteins	Clusters	Singletons

R. var. *sanguineum*	53,207	27,542	23,439
R. var. *haemaleum*	46,754	25,722	18,394
R. var. *didymoides*	38,548	25,121	12,359
total	138,509	31,525	54,192

Note: The three varieties formed a total of 31,525 clusters, of which 16,361 were orthologous clusters (contained at least two taxa) and 15,164 were single-copy gene clusters; Singletons refer to proteins that were not included in any cluster.

Table S5 Identification and annotation of orthologous clusters across three varieties of the *Rhododendron sanguineum* complex. **See attachment (TableS5.xlsx)**

Table S6 List of the 40 predicted anthocyanin-associated genes and paralogs found in the *Rhododendron sanguineum* complex.

Ortholog	gene/ paralog	RsD_01	RsD_02	RsD_03	RsH_01	RsH_02	RsH_03	RsS_01	RsS_02	RsS_03
cluster15940	*Rs4CL*	28.82	11.44	7.95	17.74	49.74	44.11	20.75	26.63	125.95
cluster16061	*RsANS1*	7.17	75.78	137.90	1263.14	99.20	84.40	124.45	94.86	58.32
cluster5445	*RsANS2*	41.84	18.20	91.92	217.53	108.97	107.49	87.19	51.63	50.94
cluster10934	*RsAT1*	2.51	149.35	69.75	36.92	73.36	38.15	26.42	0.46	1.23
cluster15359	*RsAT2*	5.87	17.65	0.42	4.42	18.38	10.41	0.00	0.00	3.06
cluster11902	*RsBGLU1*	25.15	34.13	93.20	10.38	4.22	2.54	25.24	33.82	103.01
cluster12518	*RsBGLU2*	200.44	171.79	187.23	131.13	66.93	107.48	208.25	422.64	346.80
cluster12944	*RsBGLU3*	9.56	76.65	164.20	8.19	10.15	8.14	44.29	19.28	40.73
cluster13649	*RsBGLU4*	4.33	7.07	9.65	2.83	14.40	7.43	3.07	0.13	0.69
cluster13812	*RsBGLU5*	4.04	1.29	4.67	1.58	0.35	0.14	1.11	1.27	0.81
cluster17069	*RsBGLU6*	4.74	3.04	15.86	4.26	2.45	1.87	4.64	0.99	1.64
cluster4481	*RsBGLU7*	63.56	62.95	66.82	208.71	64.21	102.44	112.17	116.54	138.05
cluster3369	*RsCAD1*	288.83	88.09	180.65	151.65	76.41	94.36	34.80	74.86	132.07
cluster6764	*RsCAD2*	39.55	36.53	32.02	21.33	14.49	39.47	9.59	15.56	14.23
cluster4247	*RsCHI1*	7.99	27.70	95.40	650.58	129.02	109.39	60.70	71.05	92.84
cluster6582	*RsCHI2*	316.37	952.32	168.71	412.10	425.60	301.14	264.63	96.43	113.28
cluster3634	*RsCHS*	0.28	0.63	3.30	75.44	5.96	17.88	4.12	225.90	0.50
cluster7213	*RsF3H*	65.14	67.68	106.21	231.88	157.14	148.44	197.14	117.69	101.28
cluster6528	*RsF3'H*	15.45	52.98	78.63	358.94	68.80	71.27	56.91	41.98	34.23
cluster11877	*RsFLS1*	0.43	13.25	6.30	48.45	23.34	15.60	1.79	1.46	3.61
cluster14248	*RsFLS2*	2.02	6.16	13.32	60.28	31.07	18.56	11.33	17.51	238.51
cluster5987	*RsFLS3*	36.56	141.76	78.70	199.55	54.36	142.26	40.97	70.89	43.31

cluster12141	*RsGST1*	1.76	159.97	1144.26	536.71	2778.92	1494.17	33.11	741.56	10.97
cluster14387	*RsGST2*	0.40	1.29	0.67	2.99	1.55	4.30	1.03	16.71	0.00
cluster15396	*RsGST3*	82.87	82.80	17.58	19.75	115.60	114.13	258.95	193.53	246.75
cluster15423	*RsGST4*	8.68	190.60	16.53	279.49	159.28	243.98	64.11	101.79	47.28
cluster17123	*RsGST5*	0.00	4.93	80.23	181.07	109.35	56.67	1.29	27.40	1.69
cluster1964	*RsGST6*	3.60	6.19	15.07	84.67	16.91	8.57	4.03	5.96	10.54
cluster3729	*RsGST7*	30.62	244.89	93.57	544.77	241.53	247.98	111.01	350.26	82.73
cluster3778	*RsGST8*	1.15	0.31	1.74	4.32	1.91	5.65	1.55	42.07	0.95
cluster9016	*RsGST9*	2.80	3.39	0.97	31.41	2.70	2.71	4.95	0.89	1.08
cluster4931	*RsOMT*	18.75	3.67	77.02	33.56	5.85	4.16	1.85	3.20	1.36
cluster12247	*RsPER1*	334.54	786.75	636.93	32.34	8.11	13.37	1099.76	2984.30	1440.03
cluster16645	*RsPER2*	0.94	40.64	1.31	81.78	18.10	231.10	12.85	16.85	10.32
cluster1906	*RsPER3*	184.28	91.20	153.42	26.49	12.66	7.65	134.26	80.33	139.30
cluster5800	*RsPER4*	40.95	22.62	42.73	16.77	11.99	22.02	14.26	70.36	65.84
cluster6367	*RsPER5*	1.93	10.72	3.18	3.33	4.90	2.15	4.81	4.85	5.87
cluster6658	*RsPER6*	0.23	0.38	14.22	0.00	0.00	0.46	3.53	6.83	1.45
cluster7193	*RsPER7*	9.80	14.26	25.08	1.93	9.18	2.33	4.33	1.67	4.50
cluster8033	*RsPER8*	4.43	11.18	12.76	3.14	7.22	3.85	31.72	38.43	20.05

Note: *GAPDH* (GenBank acc. no. FN552706) was utilized as an internal control for normalization. *BGLU*, beta-glucosidase; *PER*, peroxidase; *CAD*, cinnamyl alcohol dehydrogenase; *4CL*, 4-coumarate-CoA ligase; C*HS*, chalcone synthase; *CHI*, chalcone isomerase; *F3H*, flavanone 3-hydroxylase; *F3'H*, flavanone 3′-hydroxylase; *FLS*, flavonol synthase; *ANS*, anthocyanin synthase; *AT*, acetyltransferase; *OMT*, o-methyltransferase; *GST*, glutathione S-transferase.

Table S7 Primer information used for qRT-PCR validation of 12 gene paralogs involved in anthocyanin synthesis in three varieties of the *Rhododendron sanguineum* complex. **See attachment (TableS7.xlsx).**

Table S8 Statistical information of promoters for all genes among the *Rhododendron sanguineum* complex. **See attachment (TableS8.xlsx).**

(a)

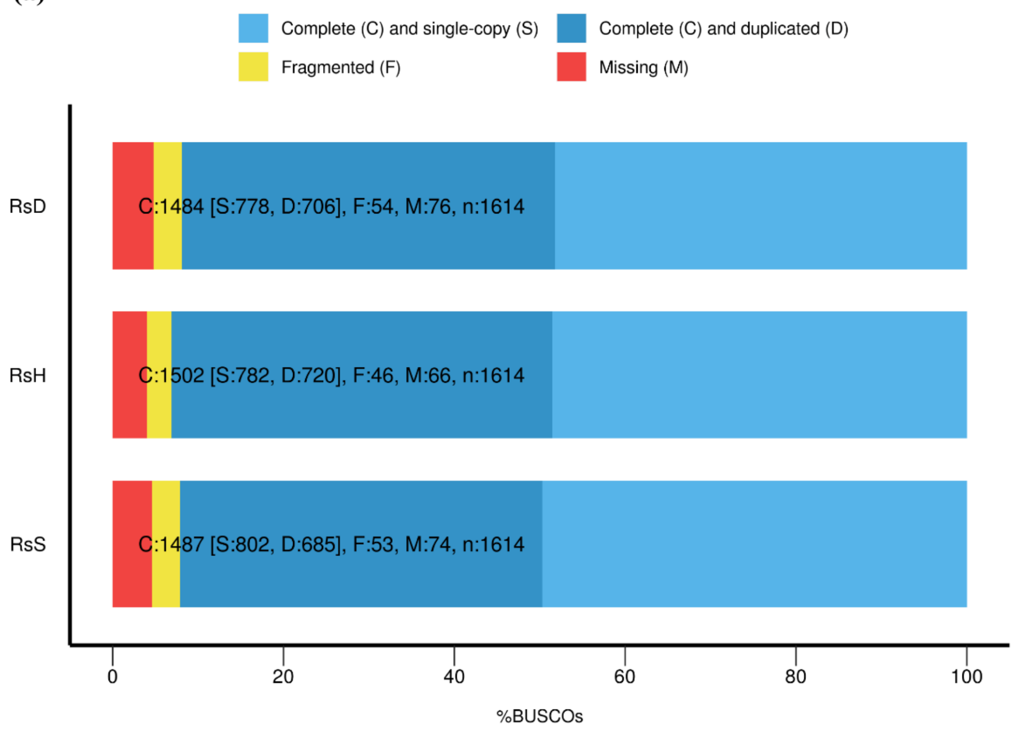

(b)

Annotated database	Number of cluster hits	Percentage (%)
NR	14,441	95.23
Swiss_prot	10,648	70.22
GO	7,445	49.10
COG/KOG	13,089	86.32
eggNOG	14,109	93.04
KEGG	4,302	28.37

Figure S1 BUSCO quality assessment results of transcriptome assemblies for three varieties of the *Rhododendron sanguineum* complex (a) and annotation of 15,164 orthologous cluster hits against six different databases (b). RsS – *R. sanguineum* var. *sanguineum*; RsH – *R. sanguineum* var. *haemaleum*; RsD – *R. sanguineum* var. *didymoides*.

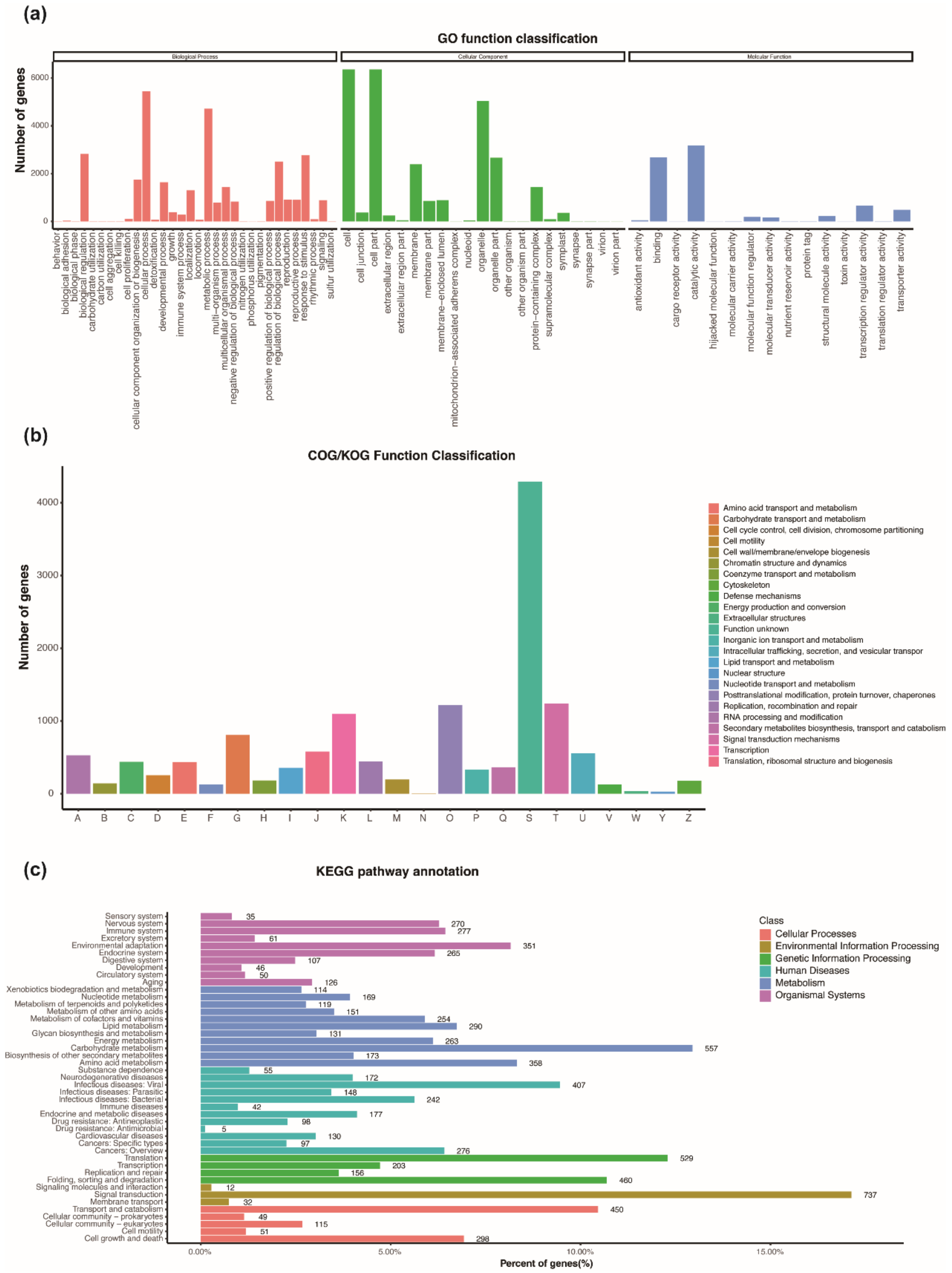

Figure S2 Annotation of the 15,164 one to one single copy orthologs among three varieties of the *Rhododendron sanguineum* complex through interrogation against three different databases. (a) Histogram of gene ontology (GO) functional classification of all 15,164 one to one single copy orthologs that were placed in three main categories: ‘biological process (BP)’, ‘cellular component (CC)’ and ‘molecular function (MF)’. (b) COG/KOG classification of the 15,164 orthologs. Capital letters on the horizontal axis indicate the COG/KOG categories, which are explained below the histogram, and those on the vertical axis indicate the number of genes. (c) KEGG pathway annotation of the 15,164 orthologs. Vertical axis shows the annotations of the KEGG

metabolic pathways, and the horizontal axis represents the gene numbers annotated in each pathway.

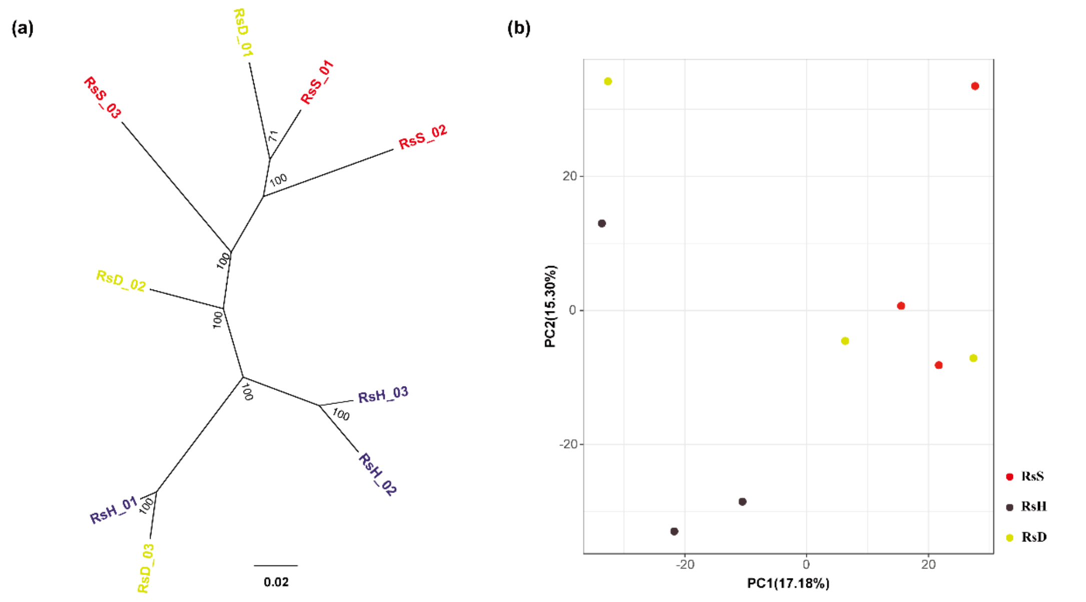

Figure S3 Phylogenetic and genetic clustering results for the three varieties of the *Rhododendron sanguineum* complex. (a) Maximum likelihood tree of 9 individuals of three varieties based on 50,853 SNPs, as implemented in SNPhylo. The samples are color coded by varieties. Node values represent the percentage values of 1,000 bootstrap replicates. The scale bar represents substitutions per site. (b) Principal component analysis of the 50,853 SNPs data set on 9 individuals of three varieties. The samples are color coded by variety according to the legend to the right of the PCA plot. Variety code as in Figure S1.

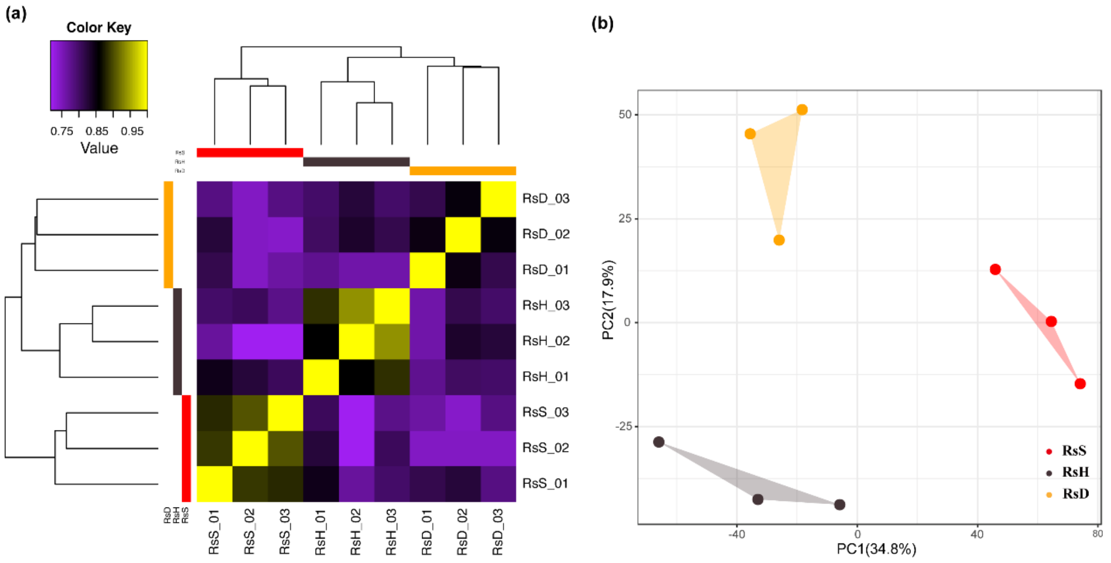

Figure S4 Gene expression patterns of all 18 samples of three varieties of the *Rhododendron sanguineum* complex. (a) Symmetrical heatmap of Pearson's correlation coefficient between all pairs of samples. (b) Principal component analysis of the log-transformed normalized expression levels across all samples. Variety code as in Figure S1.

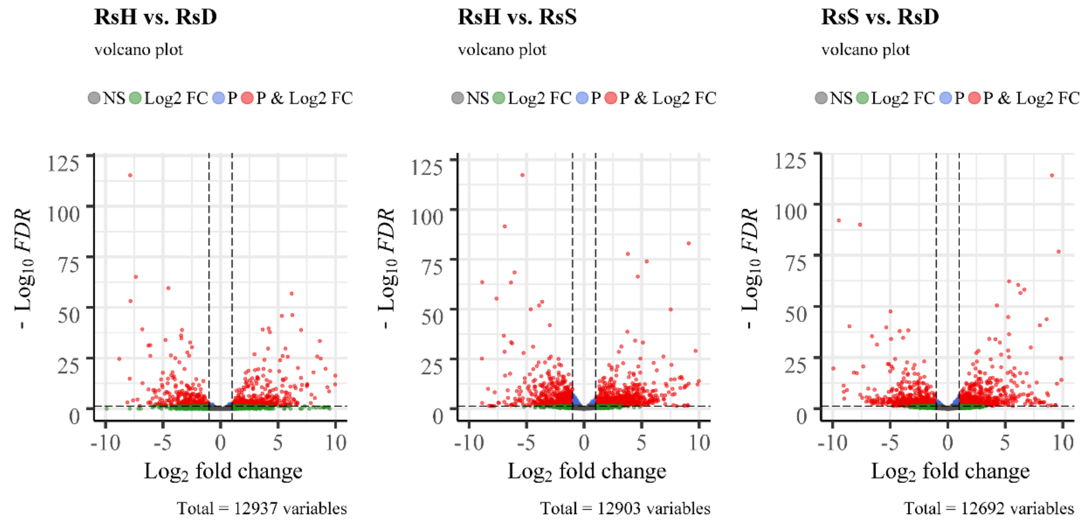

Figure S5 Volcano plots of differentially expressed genes (DEGs) based on pairwise comparisons of the three varieties of the *Rhododendron sanguineum* complex. Expression data were plotted on a log2 fold change (x-axis) versus a -log10 transformation of the *p*-adjusted values (y-axis). Each dot represents one gene. Red dots represent differentially expressed genes. Other colored dots represent genes with unbiased expression. Variety code as in Figure S1. NS – not significant genes; Log2 FC – significant log2 fold change, but not the *p*-adjusted values genes; P – significant *p*-adjusted values, but not the log2 fold change genes; P & Log2 FC – significant differentially expressed genes.

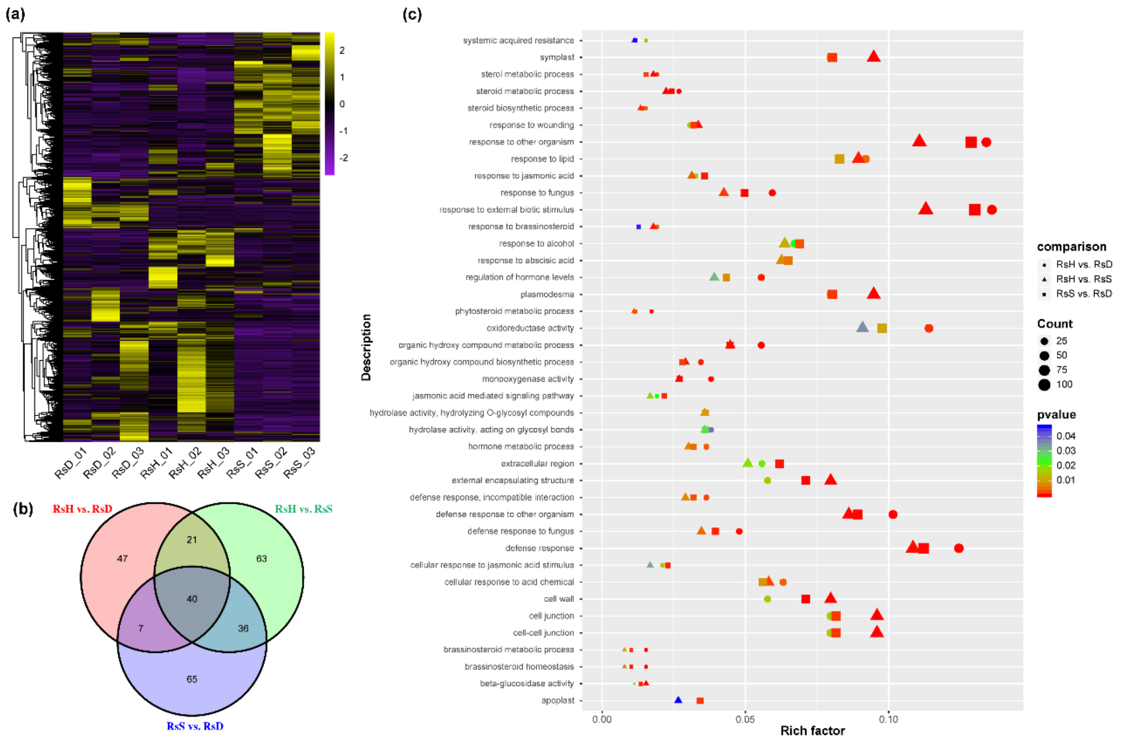

Figure S6 Heatmap and results of the functional enrichment of differentially expressed genes (DEGs) among the three varieties of the *Rhododendron sanguineum* complex. (a) Hierarchical clustering of normalized expression levels for all global expressed genes shows distinct gene expression profiles in the varieties. Yellow indicates higher while purple marks lower expression.

(b) Venn diagram the three pairwise variety comparisons. (c) Scatterplot of the 40 common GO terms functional enrichment of differentially expressed genes among three pairwise comparisons. Variety code as in Figure S1.

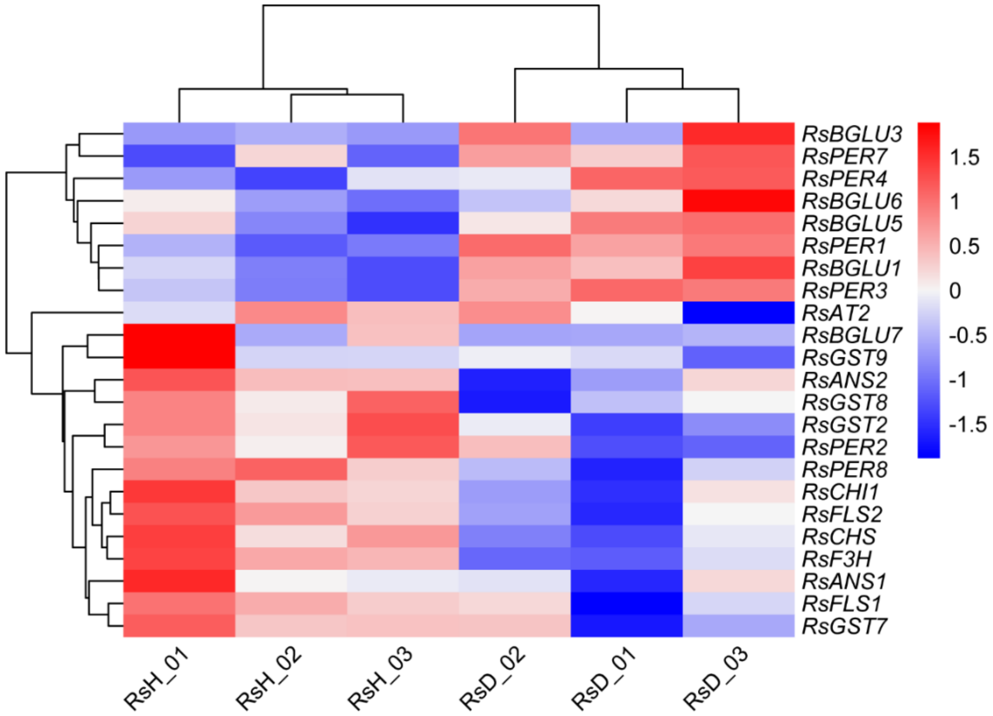

Figure S7 Hierarchical clustering of normalized expression levels of 23 candidate genes show distinct gene expression profiles in comparison of varieties *RsH* and *RsD*. Variety code as in Figure S1. Red indicates high expression, and blue indicates low expression.

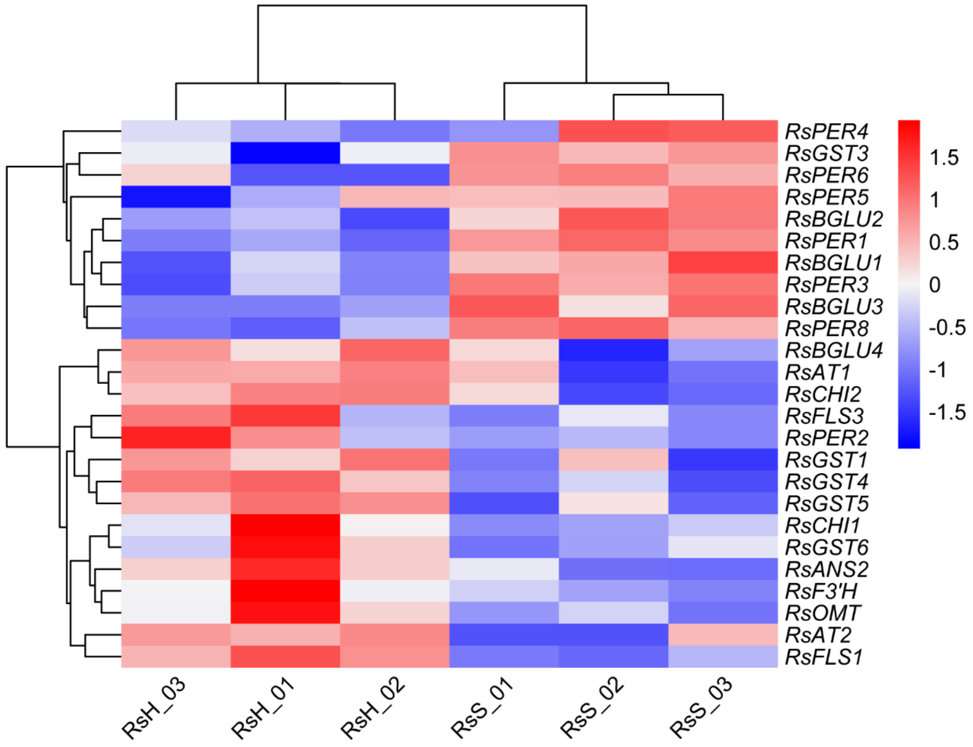

Figure S8 Hierarchical clustering of normalized expression levels of 25 candidate genes show

distinct gene expression profiles in comparison of *RsH* and *RsS*. Variety code as in Figure S1. Red represents high expression, and blue represents low expression.

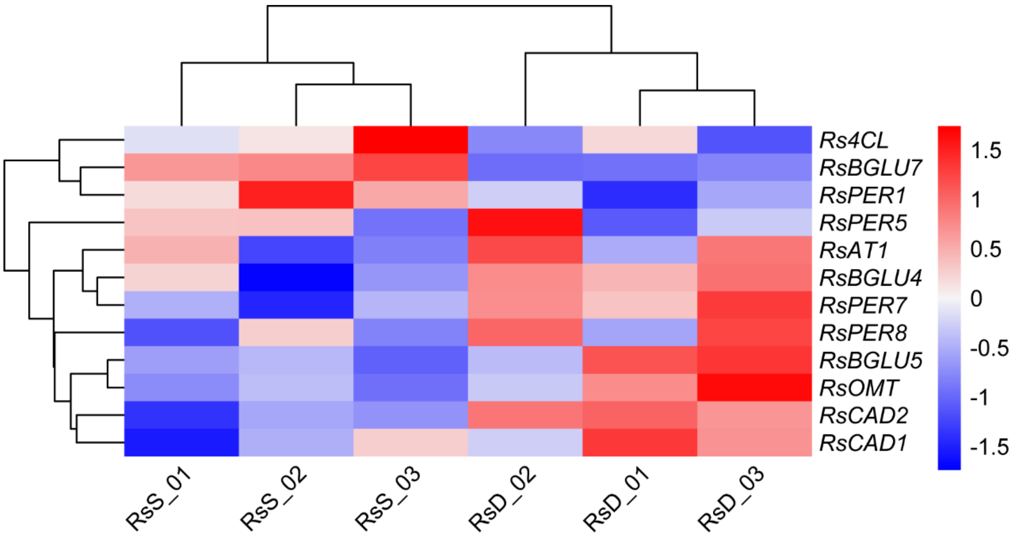

Figure S9 Hierarchical clustering of normalized expression levels of 12 candidate genes show distinct gene expression profiles in comparison of *RsS* and *RsD*. Variety code as in Figure S1. Red represents high expression, and blue represents low expression.

Figure S10 A common layout of the promoter architecture of the 13 anthocyanin genes across varieties. The sequences are 2 kb, covering the 5′-noncoding regions upstream of the translation initiation sites. The *cis*-acting elements related to the MYB-binding sites are represented by solid boxes and the variations are marked with red arrows. Graphical outputs show sequence similarity in a range of 75%-100% and GC content in a sliding window of 50 bp. Light-gray areas highlight slight sequence variations. Variety code as in Figure S1. **See attachment (Figure S10.pdf)**
